# **Primary Healthcare Policy Research: Including Variables Associated With the Social Determinants of Health Matters** Comment on "Universal Health Coverage for Non-communicable Diseases and Health Equity: Lessons From Australian Primary Healthcare"

**DOI:** 10.34172/ijhpm.2021.102

**Published:** 2021-09-05

**Authors:** Shannon Berg

**Affiliations:** ^1^Department of Occupational Therapy, Faculty of Rehabilitation Medicine, University of Alberta, Edmonton, AB, Canada.; ^2^Centre for Clinical Epidemiology and Evaluation (C2E2), Vancouver Coastal Health Research Institute, Vancouver, BC, Canada.; ^3^Department of Occupational Science and Occupational Therapy, University of British Columbia, Vancouver, BC, Canada.

**Keywords:** Health Policy, Primary Healthcare, Universal Health Coverage, Health Equity, Social Determinants of Health

## Abstract

Fisher et al have provided a solid addition to health policy literature in their finding that universal health coverage supports equitable access to Australian primary healthcare (PHC), despite factors such as episodic care and poor distribution of services. Their definition of PHC was comprehensive, extending beyond medical care to include social determinants of health and public policy. However, they limited their operational definition for purposes of the study to general practice, community health and allied health. Applying a narrower definition risks lost opportunities to identify policy implications for equity beyond financial accessibility. The populations most at risk of non-communicable diseases also face significant language, culture, and individual and systemic discrimination barriers to access. Future policy research should consider using a comprehensive PHC definition in determining variables of interest and designing research methodologies, to avoid missing important knowledge that allows existing biases within primary care to continue.

 Congratulations to Fisher et al^1^ for a significant contribution to the policy literature related to the advancement of primary healthcare (PHC). The underlying theory tested by the research is useful for policy-makers to consider – that universal health coverage is associated with health equity in PHC, particularly for non-communicable disease. The authors describe in their background a primary care system where policy-makers remain focused on driving change through reforms centred on primary care providers (primarily family physicians). Policy-makers who participated in this research mainly considered non-communicable disease interventions targeted at individual behavior change (diet, exercise, use of tobacco and alcohol) rather than population based approaches that might influence the social determinants of health. The authors found that a mixture of public and private insurance coverage contributed to unequal access and poorer health outcomes, resulting in the need for the health system to respond to increased levels of non-communicable disease.

 Fisher et al^1^ defined PHC as “comprehensive first-level care that incorporates but extends beyond medical care to include health promotion, disease prevention, community engagement and action to address the social determinants of health, and public regulation of key social determinants on non-communicable disease, such as the products and practices of tobacco, food and alcohol industries” (p. 2). They placed conditions on this definition for purposes of their research, referring to this broad definition as comprehensive PHC, and limiting their operational definition of PHC to first-level services including general medical practice, community health and allied health services.

 I acknowledge the need to limit operational definitions for the purposes of the research undertaken, however, I submit that there are risks to this approach when considering PHC. Social determinants of health represent very basic factors that lead to positive quality of life and health. PHC represents structures and processes that lead to provision of services and care that support quality of life and health. Therefore, how PHC is structured, and the processes used in a PHC system, have an impact on access and equity. In this regard, the PHC system itself can be a factor that influences the social determinants of health.^2^ Limiting the definition based on existing structures and processes may introduce a systematic bias that precludes addressing issues of equity for populations most in need of PHC services.

 While universal coverage is associated with equity, there are other equally or more important variables associated with equity. Certainly universal coverage, defined as when people receive required health services without sustaining financial hardship,^3^ is a recognized variable with regard to access to healthcare generally, and PHC in particular. In a systematic review of private compared to public health service provision in middle to low-income countries, public access was associated with better quality, patient outcomes, fairness/equity, and accountability, as well as lower cost. However, private sector services were associated with better timeliness and customer service, although they tended to target more affluent patients.^4^ Universal access achieved through a single payer system (such as a national health insurance plan) has been associated with lower per capita healthcare costs, lower rates of cost as a barrier to care, increased quality in terms of health outcomes and patient satisfaction, but lower quality with regard to wait times.^5^

 However, to consider equity in the context of access, one must broaden the definition of access beyond universal health coverage to include other factors associated with the social determinants of health. Universal coverage is not the same thing as universal access.^3^ Good access includes absence of financial barriers, which universal coverage partially addresses. In addition, it also includes physical attributes such as availability as a function of distance, timeliness and physical accessibility. Moreover, access includes factors associated with cultural and social acceptability, such as language, ethnicity, age, sexual orientation, gender, etc.^5,6^ Health inequities are comprised of avoidable variation in health status across populations.^2^ Inequity of access to health services, therefore, describes a condition of differing access to necessary health services based on characteristics of the health system that are, arguably, changeable, including but not limited to financial coverage.

 This leads logically to consideration of the social determinants of health. A Canadian study exploring inequity across populations found significantly lower indicators of health status for people in lower income brackets (income, educational levels achieved and employment status considered), Indigenous people, sexual and racial minorities, immigrants, and people living with functional limitations.^7^ Life expectancy and health-adjusted life expectancy were lower, while infant mortality and deaths due to unintentional injury and suicide were higher for these populations. There was higher prevalence of a range of chronic conditions, including mental health related issues, diabetes, arthritis, asthma and obesity. Smoking and exposure to second hand smoke was higher, and prevalence of living in substandard housing was higher. There was a higher proportion of vulnerability associated with early childhood development factors, as well as higher rates of household food insecurity.

 Yet these populations are the ones least likely to be comfortable accessing PHC services as they are currently structured. Across eleven Commonwealth countries that have universal health coverage, people living with mental health conditions, lower income, and those who were born outside of the country in which they were living were found to be more likely to face multiple barriers to accessing primary care.^6^ Even in a country that has universal health coverage, people living with low incomes may face financial barriers to PHC. People working multiple jobs may not have the time to attend appointments; they may have jobs that do not provide paid appointment and sick time; transportation options to attend appointments may be a barrier; and they may be unable to afford out-of-pocket expenses for uncovered health costs such as therapy and pharmaceuticals.^8^

 Perhaps more importantly, people may avoid accessing care, even if this places their health at risk, if they perceive that their culture and language are not respected and/or they are being discriminated against or undervalued.^8^ Language barriers exacerbate issues with health literacy, and may lead to miscommunication and, therefore, lack of optimal care.

 Providers often bring their biases and assumptions to their practice, which can lead to access barriers for patients who are already at more risk of poor health outcomes. PHC is relationship based, and in countries with a history of colonization, many healthcare providers view Indigenous people as less worthy of care based on factors such as their Indigenous status, income, housing status, or presumed substance use history. Providers’ assumptions about factors related to the social determinants of health may lead to blaming individuals for not taking responsibility for their own health, and may affect assessment and treatment decisions.^9^ In a study examining variables predictive of medication under-use (a behavior associated with PHC) across seven countries, it was found that in addition to financial issues, patient characteristics including age, ethnicity, depression and involvement in their treatment decisions were significant,^10^ all of which can be influenced by provider bias and assumptions.

 For these reasons, it is important for policy researchers to use the full definition of PHC, and consider the social determinants of health when evaluating factors related to its success. Narrowing the definition to primary and medical care may allow issues of acceptability and accessibility to fly under the radar, when sometimes they may be the most significant barriers to achieving equity.

 It may be that experiences in managing population health due to the coronavirus disease 2019 (COVID-19) pandemic provide opportunities for PHC to respond more effectively in terms of equitable access for all people. COVID-19 has had more impact on populations that live in congregate settings, have less access to healthcare, and face financial burdens such as lack of paid sick time.^11^

 Of necessity, the pandemic has resulted in healthcare systems working with community and social systems to create new ways of supporting their shared populations. Various levels of government and community agencies work together in all natural disasters to rescue and recover, while keeping the economy going,^11^ however, the partnerships are not sustained because a natural disaster has a beginning and an end, and can be contained. With the COVID-19 pandemic, Dzigbede et al^12^found that because the disaster was unseen, invisible, and can strike at any time and across multiple times, responders needed to react differently. Particularly in jurisdictions with less resources, there was a need to create and sustain partnerships that enhance community benefits. If the pandemic has resulted in sustained partnerships lasting more that a year between levels of government and community agencies that are benefitting individuals most at risk of poor health outcomes, perhaps such partnerships can be leveraged post-pandemic to change the way PHC is provided. One documented example from Canada involves primary care working together in a coordinated fashion with public health, acute care and community agencies to create and implement a comprehensive integrated pathway supporting a COVID-19 outbreak in a food processing plant that employed a significant number of temporary foreign workers and new immigrants.^13^ An opportunity for PHC policy research may be to evaluate the factors that led to more sustained partnerships created during the pandemic. New knowledge in this area could support application of the same relationships, processes and tools to other populations that require a comprehensive approach to PHC, including those that have poor access due to the social determinants of health.

 Utilizing a narrow definition of PHC, Fisher et al^1^found that despite the predominance of episodic care and poor distribution of services, universal health coverage supported equity of access to PHC in Australia. However, they may have missed opportunities to uncover access issues for people who are most in need of comprehensive PHC by not including variables that influence acceptability. They described the Australian PHC policy reforms as focused primarily on family physicians and individual behavior change, and therefore may have missed access issues related to services intended to address the social determinants of health, provider bias, and systemic discrimination. Future policy research studying effective and efficient PHC should consider the full definition of PHC in determining variables of interest and designing research methodologies. By limiting the operational definition used, researchers may miss important knowledge that allows existing biases within primary care to continue.

## Acknowledgements

 Thanks to Ashley Stacewicz for providing valuable editing and suggestions to improve clarity.

## Ethical issues

 Not applicable.

## Competing interests

 Author declares that she has no competing interests.

## Author’s contribution

 SB is the single author of the paper.

## Disclaimer

 The views and opinions expressed in this article are those of the author and do not necessarily reflect the views or policy of any organization with whom she is affiliated.
